# Endocytosis at the Crossroad of Polarity and Signaling Regulation: Learning from *Drosophila melanogaster* and Beyond

**DOI:** 10.3390/ijms23094684

**Published:** 2022-04-23

**Authors:** Fani Papagiannouli

**Affiliations:** Medway School of Pharmacy, Universities of Kent and Greenwich, Chatham Maritime, Chatham ME4 4ZF, UK; f.papagiannouli-227@kent.ac.uk

**Keywords:** cell trafficking, endocytosis, lysosome, signaling regulation, polarity, Dlg, Scrib, Lgl, EGFR, Notch, *Drosophila* testis, squamous epithelia, intestine, autophagy, PAR complex, JNK, Rab proteins, nephrocytes, SOP, *Saccharomyces cerevisiae*, Arrestins, mTOR, TORC1

## Abstract

Cellular trafficking through the endosomal–lysosomal system is essential for the transport of cargo proteins, receptors and lipids from the plasma membrane inside the cells and across membranous organelles. By acting as sorting stations, vesicle compartments direct the fate of their content for degradation, recycling to the membrane or transport to the trans-Golgi network. To effectively communicate with their neighbors, cells need to regulate their compartmentation and guide their signaling machineries to cortical membranes underlying these contact sites. Endosomal trafficking is indispensable for the polarized distribution of fate determinants, adaptors and junctional proteins. Conversely, endocytic machineries cooperate with polarity and scaffolding components to internalize receptors and target them to discrete membrane domains. Depending on the cell and tissue context, receptor endocytosis can terminate signaling responses but can also activate them within endosomes that act as signaling platforms. Therefore, cell homeostasis and responses to environmental cues rely on the dynamic cooperation of endosomal–lysosomal machineries with polarity and signaling cues. This review aims to address advances and emerging concepts on the cooperative regulation of endocytosis, polarity and signaling, primarily in *Drosophila melanogaster* and discuss some of the open questions across the different cell and tissue types that have not yet been fully explored.

## 1. Introduction

Cellular trafficking and the endosomal–lysosomal system regulate multiple developmental processes from phagocytosis, nutrient uptake and plasma membrane lipid or protein homeostasis, to signaling from cell-surface receptors and membrane trafficking [1,2]. By actively regulating cellular responses, endocytosis and vesicular trafficking play a critical role in setting up and organizing tissue formation, cell remodeling and cell communication. In doing so, endosomal–lysosomal trafficking machineries interact with polarity components and scaffolding proteins to internalize receptors from the cell membranes and target them to various specialized compartments inside the cells [3,4]. Thus, endocytosis can directly regulate the activation and termination of signaling responses, and thereby influence how cells respond to environmental cues.

Endocytosis and vesicle trafficking play an equally important role in establishing polarity by coordinating intracellular protein trafficking and the distribution of protein-sorting signals to distinct plasma membrane domains [5,6,7]. In some cell types, such as *Drosophila* neuroblasts, polarity relies more on the differential distribution of cell fate determinants, while classical epithelia polarity involves the differential localization of transmembrane and adaptor proteins or targeting of the transport machinery across apical vs. basolateral membranes [5,8,9] (Figure 3C). Reciprocally, polarized cytoskeletal, adaptor and scaffolding proteins participate in binding and directing endosomal, membrane trafficking components and signaling platforms or exocytic delivery to specific subcellular domains. For example, restriction of endocytosis to micropatterns, defined by cell adhesion geometry, can define the topology of signaling reception and downstream propagation [10]. Although endocytosis cooperates with cytoskeletal and adhesion proteins, to direct the polarized distribution of receptor-containing vesicles inside the cells, in many cases, localized signaling reception can also define the subcellular delivery of endocytic and cargo-sorting events [5]. Therefore, the interplay of endocytosis with polarity and signaling seems to be highly dynamic and context-dependent.

Research over recent years supports this view of a complex system in which endocytosis, polarity and signaling are intimately linked in multidirectional regulatory networks that depend on the cell-type specific availability of components across different tissue systems [3,4,11]. Uncovering the underlying mechanistic and functional principles of intracellular trafficking, polarity and signaling across various tissue systems and organisms has shed light on the plethora of interactions and the multiple levels of control that orchestrate tissue morphogenesis and homeostasis [11,12].

## 2. Endocytosis in Signaling Regulation: Setting the Stage

**Figure 1:**
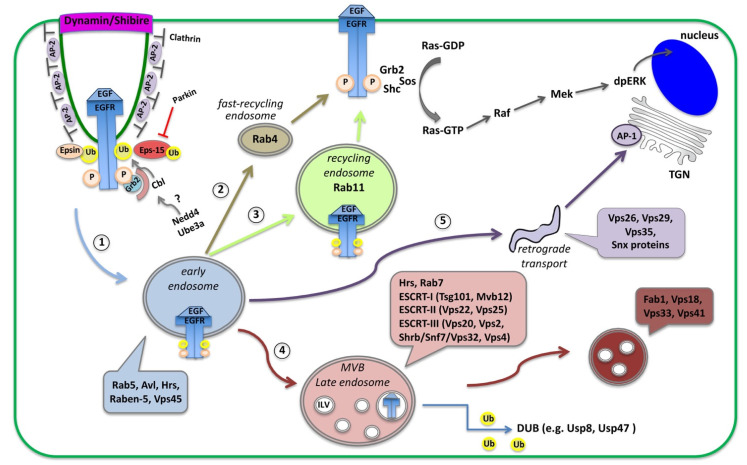
Schematic diagram of receptor endocytosis and trafficking routes within the cells. Here, the EGFR is shown as an example of receptor endocytosis, along with adaptor proteins recruited upon binding of the EGF ligand. Activation of the EGFR initiates the Ras/MAPK cascade that leads to double phosphorylation of the MAPK (dpERK), which translocates into the nucleus and activates transcription. Activated EGFR is removed from the plasma membrane via clathrin-mediated endocytosis (CME). Receptors loaded on Rab5-containing early endosomes (1), can follow alternative routes and (2) recycle back to the membrane by a Rab4 fast recycling endosome, (3) traffic to Rab11-containing recycling endosome, (4) proceed to multi-vesicular bodies (MVB), late endosomes and lysosomes for degradation, or (5) traffic to sorting endosomes or to the trans-Golgi network (TGN) via retromer trafficking.

Signaling is critically required for cell-coordinated function and for setting up complex tissues. Cells communicate with each other through the activation of receptors located at their surface plasma membrane. However, the fine trimming of signaling levels is also critical for proper cellular output and maintaining homeostasis. Signal attenuation is based on the internalization and removal of activated ligand-receptor complexes from the membrane via endocytosis [13,14,15]. Endocytosis of activated receptors follows clathrin-mediated endocytosis (CME) [13,16,17] but can also follow clathrin-independent pathways, e.g., caveolar-type (reviewed in [18,19,20,21]). In CME, activated receptors are recruited to clathrin-coated pits by interacting with the adaptor protein (AP)-2 complex, then clathrin-coated pits invaginate and pinch off with the action of the GTPase Dynamin, encoded by the *shibire* gene in *Drosophila* [13,17,22] (Figure 1). Another constituent of clathrin-coated membranes is the membrane phospholipid Phosphatidylinositol 4,5-bisphosphate [PtdIns(4,5)P2] (PIP2), that binds and recruits AP-2 [16].

Once loaded on endosomes, internalized receptors can be either recycled back to the cell surface or transported to lysosomes for degradation. Initially, receptor-containing vesicles fuse to the early endosome (EE) where endosomal trafficking is controlled by the Rab proteins, small GTP-binding proteins of the Ras superfamily. Each Rab protein resides in a particular type of endosome and recruits specific effector proteins. EEs, containing Rab5 and the syntaxin7 Avalance (Avl) [23], can follow several alternative routes: (1) rapidly recycle back to the membrane by a Rab4-dependent mechanism; (2) traffic to the Rab11-containing recycling endosome (RE) to recycle to the plasma membrane; (3) proceed to multi-vesicular bodies (MVB), late endosomes (LE) and lysosomes for degradation; or (4) traffic to the *trans*-Golgi network (TGN) in the so-called retromer trafficking or to sorting endosomes that mediate apical or basolateral trafficking [24,25] (Figure 1). MVBs are defined by the presence of intra-luminal vesicles (ILV) that are formed in a process of inward membrane invagination involving the Endosomal Sorting Complex Required for Transport (ESCRT) complexes. The formation of ILV within MVBs is a critical step in signaling regulation. Before delivery into the ILVs, receptors can still bind their ligands and continue signaling through their cytoplasmic domain. Internalization of receptors into the ILVs segregates their intracellular domain from the cytoplasm and terminates signaling [26,27]. Maturation of EE to LE involves the acquisition of Rab7 and loss of Rab proteins that are involved in receptor recycling (Figure 1). Interestingly, in many cases receptor internalization is important not for silencing the receptor but to transport the ligand-receptor complexes into the cell for signal maintenance or propagation in endosomal compartments that act as signaling platforms [15,28,29].

## 3. EGFR Signaling: Activation, Trafficking and Physiological Importance across Systems

Epidermal Growth Factor Receptor (EGFR) is a receptor tyrosine kinase (RTK), which upon ligand binding, it dimerizes, becomes phosphorylated and a complex of signaling molecules assembles and initiates the downstream MAPK/Ras signaling cascade [14,30]. EGFR phosphorylation leads to the recruitment of signal-transducing adaptor proteins Grb2, the *Drosophila* Downstream of Receptor Kinase (Drk), and SHC-adaptor protein (Shc) [17,31,32], which allow the assembly of signaling complexes on the cytoplasmic tail of the activated EGFR. Grb2 recruits Sos (Son of Sevenless) but Shc is also able to link the Grb2-Sos complex to the activated EGFR. Sos stimulates Ras activation and initiates the MAPK cascade with a series of activation events that lead to double phosphorylation of the MAPK dpERK, which translocates to the nucleus and activates transcription [21,30,33] (Figure 1).

At the same time, EGFR phosphorylation activates the recruitment of the adaptor proteins that initiate the endocytic removal from the membrane. This process is seen in several tissues and organs across species, and deregulation of this process is implicated in cancer initiation and progression [13,14]. EGFR becomes endocytosed mainly by clathrin-mediated endocytosis (CME), which happens at both low and high EGFR physiological doses [12,13,16,17,34,35]. High-saturated doses of EGF ligands induce in parallel clathrin-independent endocytosis (CIE) of the EGFR [13,21,35,36]. The membrane phospholipid PtdIns(4,5)P2 (PIP2) could potentially link endocytosis to the EGFR signaling as it not only recruits AP-2 to clathrin-coated pits, but also physically binds the EGFR juxtamembrane domain and enhances EGFR phosphorylation and activation in many tissues [37,38,39]. Moreover, the adaptor proteins Epsin and Eps15 (epidermal growth factor substrate 15) link the activated EGFR to the clathrin coat by binding to Clathrin and AP-2. Once inside the cell, EGFR-containing vesicles can recycle back to the cell surface or get transported to lysosomes for degradation or even to other parts of the cells via sorting endosomes [12,24,35].

Besides phosphorylation, ubiquitination is another critical post-translational modification for EGFR endocytosis, receptor trafficking and signaling downregulation. Ubiquitin is a highly conserved 76 amino acid polypeptide that becomes covalently linked to protein substrates including receptors [40]. The ubiquitin-activating enzyme (E1), ubiquitin-conjugating enzyme (E2) and ubiquitin ligase (E3) drive ubiquitination through the addition of a small ubiquitin (Ub) protein to the EGFR (EGFR-Ub). Mono-ubiquitination of receptor Lysine (Lys) residues, affects receptor internalization and multivesicular-body (MVB) sorting, while poly-ubiquitination (also on Lys residues of the Ubiquitin) directs proteins to degradation by the proteasome [40,41]. Thus, ubiquitin modification typically downregulates the targeted receptors and protein substrates. In *Drosophila*, Cbl (the homologue for Casitas B-lineage Lymphoma proto-oncogene) is the major E3 ligase and a negative regulator of EGFR signaling. E3 ligases of the Cbl family are major regulators of the EGFR pathway, acting both as ubiquitin ligases and multiadaptor molecules [13]. Cbl E3 ligases, are RING finger containing ubiquitin ligases that mediate a direct transfer of ubiquitin to the substrate, functioning as a scaffold to orient the ubiquitin-charged E2 with respect to the substrate protein [13,42]. Once recruited to active EGFRs, Cbl gets phosphorylated and ubiquitinates the EGFR [13,17,29,43]. Alternative splicing of Cbl in *Drosophila* creates (1) a long CblL form, specific to EGFR that binds the *Drosophila* Drk, and (2) a shorter CblS form that regulates EGFR but primarily acts on Notch endocytosis [33,44,45]. Therefore, Cbl and Eps-15 are both loaded onto the internalized EGFR endosomes.

Besides the ubiquitination of the receptor, the CME of the EGFR is also regulated by ubiquitination of its adaptors proteins, further complicating the picture [13]. Epsin and Eps-15 contain ubiquitin interacting motifs (UIMs) that allow them to bind the ubiquitinated forms of EGFR but also become ubiquitinated by E3 ligases. Eps-15 ubiquitination is performed by the Nedd4 family and Parkin E3 ligases via distinct mechanisms [46]. Once Nedd4 is self-ubiquitinated it can bind the Eps-15 UIM2 domain and ubiquitinate Eps-15. Nedd4 ligases contain tryptophan-tryptophan (WW) motifs and a HECT (homologous to the E6AP carboxyl terminus) domain that catalyzes ubiquitin addition through a two-step reaction: ubiquitin is first transferred to a catalytic cysteine on the E3 and then from the E3 to a Lys on the target substrate [13,42,47]. Besides Eps-15, Nedd4 E3 ligases are also involved in EGFR endocytosis by ubiquitinating the EGFR or Cbl [13,17,43]. Parkin is an E3-ubiquitin ligase of the RBR (RING-betweenRING–RING) family that catalyzes ubiquitin transfer through a two-step reaction (ubiquitin is first transferred to a catalytic cysteine on the E3 and then to the substrate) [42,47]. Parkin contains a ubiquitin-like (UBL) domain, which binds to the UIMs of Eps15 [46]. Ubiquitinated Eps-15 can no longer bind the EGFR and promote its endocytosis and the EGFR signaling remains active [43,48]. Thus, Parkin negatively regulates EGFR endocytosis by ubiquitinating Eps-15 [43,48]. Taken together, E3 ligases negatively regulate the substrate proteins they target, but how this affects the EGFR signaling is context-dependent.

In the endosomes, ubiquitin serves as a molecular signature for recognition by numerous endocytic adaptor proteins and for trafficking the EGFR cargo to MVBs and lysosomes for degradation. EGFR-Ub present on REs moves to MVBs and is recognized by the ESCRT-0 component Hepatocyte growth factor regulated tyrosine kinase substrate (Hrs), which can also bind Ub, and facilitate the recruitment of ESCRT-I, ESCRT-II and ESCRT-III onto the MVB membranes (Figure 1) [49]. EGFR-Ub is finally internalized in ILVs inside the MVBs via the action of ESCRT-III, which promotes the inward membrane invagination of MVBs. Interestingly, EGFR molecules impaired for ubiquitination cannot be degraded, are poorly incorporated into MVBs and are recycled back to the plasma membrane [12,13,29]. Therefore, EGFR-Ub needs to be maintained until ILV formation. Nevertheless, before entry into ILVs, EGFR needs to be de-ubiquitinated with the help of de-ubiquitinating enzymes (DUBs), which regulate the recycling of ubiquitin, as well as the Hrs and Eps-15 turnover. The available literature on EGFR agrees that EGFR-Ub in endosomal compartments is fully functional and can still signal until the ubiquitin modification is removed by DUBs. The precise timing of de-ubiquitination and whether it happens before or after the ubiquitin-dependent EGFR-ESCRT interaction remains to be elucidated [17]. Results from different tissues and organisms have led to variable results and the possibility of a tissue-specific timing cannot be excluded.

Along this line of evidence, loss of ESCRT components in some tissues results in increased EGFR signaling while in others in EGFR downregulation [11,14,17,50]. In yeast and human HeLa cells, the loss of ESCRT-I and -II sustains EGFR signaling but the loss of ESCRT-III components does not [14,51,52]. In *Drosophila* eye discs, the loss of ESCRT-I, -II or -III components leads to increased EGFR signaling, probably from endocytic vesicles [50,53]. Interestingly in imaginal discs, the Vacuolar protein sorting 4 (Vps4) promotes Epidermal growth factor receptor signaling independently of its canonical role in receptor degradation (and ILV formation) as part of the ESCRT-III complex. Vps4 performs this function by acting at the level of the receptor through an endocytosis-independent mechanism and in a tissue-specific way, since, in ovarian follicles, Vps4 is involved only in EGFR degradation [26].

On the other hand, the loss of ESCRT-0 components, such as Hrs, leads to the recycling of the EGFR receptor, which is not trapped in MVBs [11,17]. In developing imaginal discs, the ESCRT-0 components Hrs and Signal transducing adaptor molecule (Stam) play a role in silencing the EGFR by affecting the secreted form of Spitz (sSpi) [54]. EGFR signaling activation is mediated by the membrane-tethered ligand Spitz (mSpi), which requires processing by the membrane protease Rhomboid to form the sSpi. Besides its effect on Spi, Rhomboid can also cleave Star, which also mediates the post-transcriptional processing of Spi [33]. Mutations in *stam* and *hrs* cause the accumulation of Rhomboid in abnormal endosomal compartments and silence the EGFR before ligand binding [54].

Several pieces of evidence show that the EGFR-containing endosomal compartments can follow different or alternative trafficking routes depending on the specific cell type, the concentration of the ligand, the developmental stage and the cellular conditions [11,17]. In *Drosophila* trachea, EGFR signaling levels control the length of the tracheal tubes, by regulating the organization of endosomes in which Crumbs and Serpent proteins are loaded. EGFR loaded on those endosomes acts as a critical hub for the correct delivery of Crumbs (mediating apical membrane growth) and Serpent (modifier of the apical extra-cellular matrix) to their final destinations [55]. Moreover, EGFR is involved in polarity establishment by activating cell polarity regulators beyond the canonical Ras/MAPK pathway, such as the liver kinase LKB1 in *Drosophila* follicle stem cells [56]. The polarized distribution of EGFR is also crucial since, in many polarized epithelial cells, EGFR is localized primarily at basolateral sites [57,58,59]. Analysis of the retinal pigmented epithelium (RPE) in mice, revealed the critical role of the βA3/A1-crystallin in CME of the EGFR and the organization of the actin apical network [60]. βA3/A1-crystallin maintains the PIP2 pool in the RPE by attenuating the PLCγ signaling. This activates Ezrin phosphorylation and promotes EGFR internalization, which affects RPE cell polarity [60]. Another mechanism uncovered in plasmatocytes (the *Drosophila* macrophage-like hemocytes), revealed differential endocytosis of the EGFR based on ligand levels driving EGFR receptor activation [61]. More precisely, at high (but not low) levels of the ligand Spi, the EGFR is internalized in a clathrin-independent way via Graf (GTPase regulator associated with focal adhesion kinase), which is part of the GPI-enriched endocytic compartment (GEEC) endocytosis pathway. Graf interacts with the EGFR in a ubiquitination-dependent manner and promotes EGFR degradation and signaling attenuation [61].

In HeLa cells, analysis of EGFR internalization following activation with different ligands has shown that EGFR endocytosis after treatment with all ligands could be inhibited to a certain degree by ablation of clathrin, which confirms the existence of an alternative CIE pathway [62]. However, knockdown of clathrin could fully inhibit EGFR degradation with all ligands tested. The inhibition of dynamin function blocked EGFR internalization after stimulation by any of the ligands, suggesting a dynamin involvement in both CME and CIE pathways. Finally, knocking down a number of clathrin-independent dynamin-dependent pathways of internalization had no effect on the ligand-induced endocytosis of the EGFR [62].

Taken together, EGFR endocytosis and subsequent trafficking through endocytic organelles, form a dynamic network of subcellular compartments, which actively control the timing, amplitude and specificity of the signaling. Furthermore, the distribution of EGFR in apical and/or basolateral cell surfaces can have important biological consequences that affect EGFR downregulation efficiency and endocytic turnover, and influence paracrine vs. autocrine activation by different EGF ligands [35,57,58,59,63]. It would be interesting to understand how adaptor proteins and signaling complexes, often called “signalosomes”, find their subcellular way and how cortical and scaffolding proteins regulate the accessibility or subcellular trafficking of these proteins. Although a great number of elegant biochemical studies have identified binding partners and adaptor proteins involved in these processes, a lot of questions on the critical switch between EGFR recycling vs. degradation, the decisive timing of EGFR phosphorylation and ubiquitination, and its physiological importance in a cell-type-specific way, remain open.

## 4. Notch Signaling: Endosomal–Lysosomal Sorting and Polarization in Canonical and Non-Canonical Pathways

The cooperative action of endocytosis and polarity in signaling regulation is very well-illustrated in the case of Notch signaling. Research over the past decades not only revealed the regulatory patterns and alternative pathways of Notch regulation but shed light on the diversity of these networks in specific cell and tissue contexts, and across organisms (reviewed in [64,65,66,67,68,69,70,71]). Notch is a single-pass transmembrane receptor involved in cell fate decisions, morphogenetic changes (such as cell intercalation) and crosstalk with other signaling pathways [45,72,73,74,75,76,77,78,79,80].

### 4.1. Endocytosis in the Canonical Model of Notch Activation

In the canonical model, Notch binds one of the membrane-bound ligands Delta (Dl), Serrate (Ser) or Lag2 (the DSL family) from the neighboring signaling cell. This interaction initiates a cascade of proteolytic cleavages by γ-secretase that releases the intra-cellular domain of Notch (NICD). NICD can now translocate to the nucleus and activate transcription together with the transcription factor Suppressor of Hairless (Su(H)) and the nuclear effector Mastermind [81,82,83]. Although several factors that regulate Notch signaling attenuation and lysosomal degradation have been identified, internalization and endosomal sorting of Notch-NICD typically leads to activation of the receptor [50,81].

**Figure 2:**
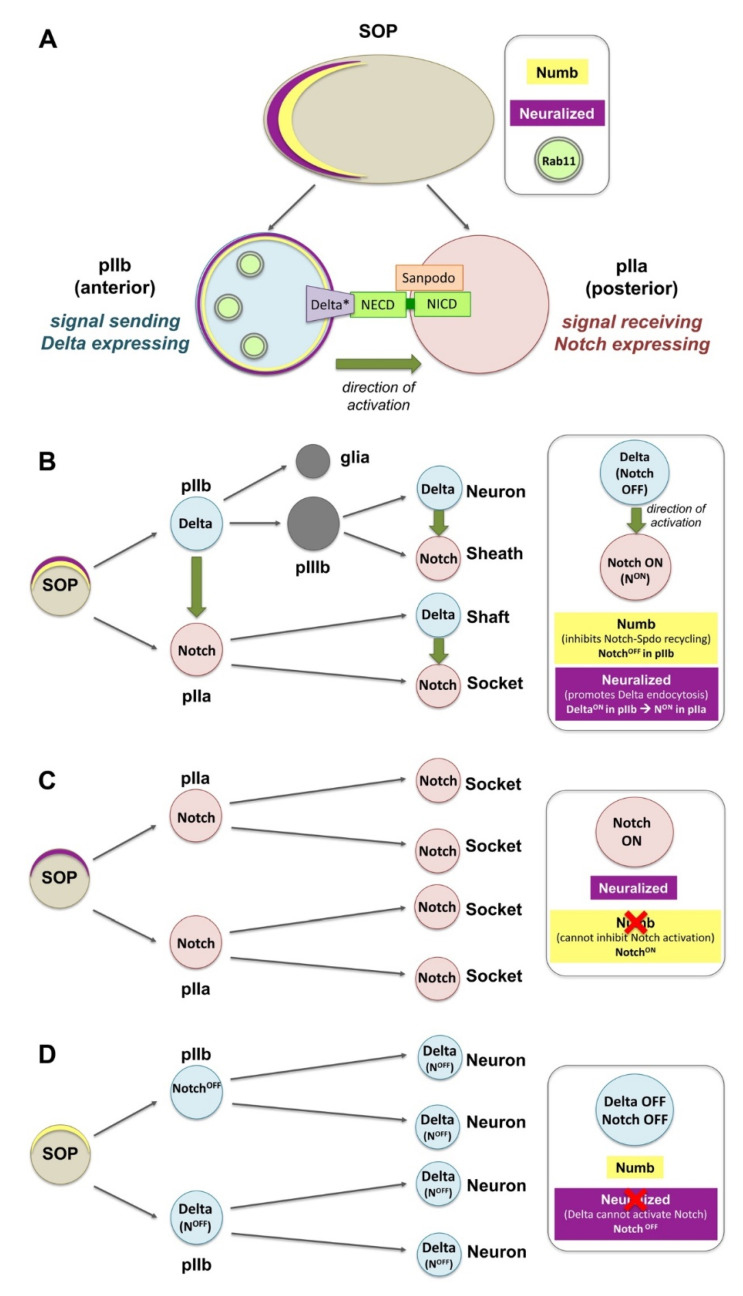
Notch activation and asymmetric distribution of fate determinants in *Drosophila* sensory organ precursors (SOPs). (**A**) The differential activation of Notch relies on the asymmetric distribution of the cell fate determinants Neuralized (Neur) and Numb in the anterior side of the SOP. Upon division, Neur and Numb get segregated into the pIIb. Numb inhibits the recycling of Notch and Sanpodo (Spdo) directing them to degradation. Neur promotes the ubiquitination and endocytosis of Delta, and thereby its activation (Delta*). Delta* recycles to the membrane via Rab11 recycling endosomes, so that it can bind the extracellular domain of Notch (the internalization events for Notch, Spdo and Delta are not shown in this diagram). (**B**) SOPs are polarized epithelial cells that divide asymmetrically to give rise to a posterior pIIa and anterior pIIb. The latter divide further to generate a neuron, a sheath, a shaft, and a socket. (**C**) Loss of *numb* can no longer inhibit Notch-Spdo recycling in the anterior pIIb where Notch now stays active, and all cells adapt the socket fate. (**D**) Loss of *neur* can no longer activate Delta in pIIb, Notch is not activated in the pIIa and all cells give rise to neurons.

Canonical Notch activation via Delta is involved in the asymmetric cell division of the sensory organ precursors (SOPs) in the pupal notum of *Drosophila*. SOPs are polarized epithelial cells that divide asymmetrically to give rise to two daughter cells, the posterior pIIa and anterior pIIb, which in turn divide asymmetrically to generate the four cells that will form the sensory organs: a neuron, a sheath, a shaft, and a socket [64,65,66,67] (Figure 2A,B). The differential activation of Notch relies on the asymmetric distribution of the cell fate determinants Neuralized (Neur) and Numb in the anterior side of the SOP and upon division, they get segregated into the pIIb (Figure 2A,B). Neur promotes the ubiquitination and endocytosis of Delta and thereby its activation (Delta*; Figure 2A). Numb inhibits the recycling of Notch and its transmembrane co-factor Sanpodo (Spdo) towards the plasma membrane (promoting their degradation). Activated Delta through Rab11-positive endosomes recycles back to the apical cell surface to activate Notch in the pIIa cell. Thus, pIIb turns off Notch, while the pIIa cell (in the absence of Neur and Numb) activates Notch and Spdo facilitates the reception of the signal. Loss of *numb* can no longer inhibit Notch-Spdo recycling in the anterior pIIb, Notch gets activated in the anterior precursor cell which adapts the pIIa fate, and cells finally adapt the socket fate (Figure 2C). Conversely, loss of *neur* can no longer activate Delta in pIIb, and Notch cannot be activated in pIIa, which now adapts the pIIb fate, and all cells eventually become neurons (Figure 2D) [64,65,66,67].

The regulated trafficking of Notch and Spdo plays an important role in this process. Numb interacts with internalized Spdo-Notch oligomers at sorting pIIb endosomes and inhibits the recycling of Notch, thereby creating an asymmetry in Notch distribution along the pIIa–pIIb interface [84]. Numb also controls the Notch receptor targeting the Rab7-containing late endosomes, a specific subpopulation of Notch endosomes. In *numb* mutants, the increased numbers of Notch signaling endosomes recycle to the membrane in the Rab11-dependent way [85]. Basolateral localization of Notch in the SOP is also regulated by AP-1 and the chaperone Stratum that follows two parallel transport routes [83]. Loss of their function leads to Notch enrichment at the apical side of the pIIa–pIIb interface. Moreover, Numb interacts with AP-1 to regulate the basolateral recycling of Spdo, while loss of *numb* permits Spdo internalization and recycling back to the plasma membrane [86]. On the other hand, phosphatidic acid (derived from Phospholipase D) promotes ectopic Notch signaling by increasing Notch endocytosis and inhibiting Sanpodo trafficking towards acidic endosomes [87].

The Actin-related protein (Arp) 2/3 complex and its activators, Scar/WAVE and Wiskott–Aldrich Syndrome protein (WASp), promote actin polymerization and influence cell shape and motility. During SOP cytokinesis, Arp2/3 and WASp are required for the recycling of Delta [88]. On the other hand, the Arp2/3 activator SCAR regulates contact expansion between pIIa and pIIb. Efficient endocytosis of Delta via the pushing force of WASp-Arp2/3 [88], is consistent with the “pulling force” model for Notch activation that exposes the buried cleavage site of the extracellular Notch to eventually produce the NICD [64,65,89].

### 4.2. Endocytosis in the Ligand-Independent Model of Notch Activation

Notch also participates in a ligand-independent, noncanonical activation through the activity of the ring finger ubiquitin ligase protein Deltex (Dx), which transports the Notch receptor from the cell surface towards the late endosomes [70,71,81,82]. Mono-ubiquitination of NICD by Dx blocks transport to MVB/ILVs and stabilizes NICD on maturing endosomes (the limiting membrane of the late endosome), which leads to Notch activation. Dx can also form a protein complex with the β-Arrestin Kurtz and the NICD. This complex switches NICD from mono- to poly-ubiquitination and targets Notch for degradation [70,71,81,82,90]. Dx-mediated Notch activation is counteracted by the function of another ubiquitin ligase Suppressor of deltex (Su(dx)), which binds and internalizes NICD in clathrin-independent endocytosis (CIE) and promotes Notch transfer to ILVs of MVBs, late endosomes and degradation. Thus, Su(dx) and Dx compete with each other in order to direct Notch through these alternative routes in a context-dependent way [71,82]. Mutations in components of the endosomal–lysosomal sorting machinery were shown capable of triggering non-canonical signaling [8,27,53,65,90]. For example, Shrub, a core component of the ESCRT-III complex, is a key regulator of the Dx and Kurtz interaction that promotes NICD endosomal/lysosomal degradation and delivery to MVBs [81]. Shrub antagonizes Dx, enhances Kurtz activity and promotes the polyubiquitinated state of the Notch. However, it is the Notch mono- vs. poly-ubiquitination state that will determine the activation vs. degradation of Notch.

Other interesting studies shed light on how adaptor, scaffold or polarity determinants mediate the crosstalk between trafficking and junctional complexes as signaling centers. One example is Polychaetoid (Pyd), a negative regulator of the Dx-mediated Notch activation in the *Drosophila* ovary stem cell niche and S2 cells [82]. Pyd is the single *Drosophila* homologue of the scaffolding zonula occludens-1 (ZO-1), junctional proteins that localize at tight junctions (TJs) in mammals. Pyd can bind and reduce Dx-dependent Notch trafficking, and thereby attenuate Notch signaling. Another interesting mechanism linking Notch to adaptor junctional components comes from the role of Crumbs in limiting ligand-independent endocytosis of Notch activation. In *Drosophila* developing wings, the apical polarity determinant Crumbs binds Notch directly and prevents its activation through the non-canonical Dx-pathway. Crumbs exerts its function by directly binding and regulating Notch localization. Yet, this function is independent of the role of Crumbs in the localization of apical components as in other tissues [82]. Taken together, Notch regulation is a very good example of the plethora of interactions where endocytosis and polarity networks converge with cell architecture and cell interactions to regulate signaling reception and recycling of Notch in order to fine-trim physiological signaling levels and cell fate decisions.

## 5. Dlg, Scrib and Lgl: Basolateral Components at the Center of Endocytosis and Signaling Regulation

### 5.1. Multitasking Polarity and Scaffolding Components

Discs large (Dlg), Scribble (Scrib) and Lethal (2) giant larvae (Lgl) are highly conserved polarity and scaffolding proteins [91,92,93,94,95,96], ranging from yeast to mammals including humans (reviewed in [97,98,99,100]). Their function has been typically studied in *Drosophila* columnar epithelia since their mutations lead to tumor formation and neoplastic transformation that captured the research interest for many years [92,93,101,102,103]. Dlg and Scrib are PDZ-containing proteins, involved in protein–protein interactions that localize at the cytoplasmic side of septate junctions (SJs) (the equivalent of vertebrate Tight Junctions) [92,99,104]. SJs build up permeability barriers in various tissues such as imaginal discs, testes, heart and sub-perineurial glial cells of the nervous system including the *Drosophila* testis [105,106,107,108]. Although, Dlg and Scrib are often referred to as “SJ proteins”, they are not part of the highly stable core protein complex that builds the SJs in *Drosophila* but are rather required for SJ localization [104]. Lgl is a cytosolic protein containing two WD40 motifs that bind to non-muscle myosin II and the cytoskeleton [109,110]. Similarly, Lgl is involved in protein–protein interactions along the basolateral portion of the plasma membrane of epithelial cells [109,111,112].

These highly conserved proteins are critically required in polarity establishment and maintenance: the basolateral Dlg, Scrib and Lgl, collectively called the Dlg-module, work together with the PAR- (Bazooka/Par3, Par6, aPKC) and the Crumbs–polarity complexes to control polarity in epithelial cells and neuroblast asymmetric cell division in finely balanced cooperative and antagonistic interactions [97,98,99,110]. Lgl homologues genetically interact with PAR components to partition cell fate determinants in *Xenopus*, MDCK (Madin-Darby canine kidney) epithelial cells and *C.elegans*. Other critical functions of the Dlg polarity module in *Drosophila* include planar cell polarity, anterior-posterior patterning of ovarian follicle cells, formation of synapses and neuromuscular junctions (NMJs) together with other scaffolding, cell adhesion or receptor complexes (reviewed in [98,100,113,114]).

The subcellular localization of these proteins and shuttling from the basolateral cortex to other cellular compartments is influenced by their phosphorylation state. Phosphorylation of Dlg by the kinases Par-1 and Calcium/calmodulin-dependent protein kinase II (CaMKII) has been shown to affect cortical Dlg targeting [115,116,117]. Dephosphorylation promotes postsynaptic recruitment of Dlg in the neuromuscular junctions (NMJs), while phosphorylation releases Dlg from its postsynaptic targeting [115,116,117]. Phosphorylation of Lgl conserved Serine (Ser)-residues by atypical protein kinase C (aPKC) (in all three Ser-residues) or Aurora A and B kinases (in 1st and 2nd Ser-residues) releases Lgl from its binding to non-muscle myosin II (encoded by the *zipper* gene in *Drosophila*) and the cortex, and promotes binding to the GUK domain of Dlg [96,109,110,118,119]. Thus, Dlg, Scrib and Lgl emerge as dynamic multitasking polarity and scaffolding components that recruit proteins to specific subcellular membrane surfaces, organize, and stabilize supramolecular adhesion and signaling complexes.

### 5.2. Dlg, Scrib and Lgl as Signaling Regulators

Dlg, Scrib and Lgl cooperate with signaling pathways in normal situations and in cancer. *dlg*, *scrib* or *lgl* mutant animals typically develop tumors, whereas knocking down any of these genes in discrete clonal patches surrounded by normal tissue, leads to cell competition that eliminates the tumorous clones. Elimination of these clones is mediated by Jun N-terminal kinase (JNK)-dependent cell death from the surrounding healthy microenvironment [113,120,121,122,123,124]. Blocking apoptosis in these clones via p35 or Death-associated inhibitor of apoptosis 1 (Diap1) expression cannot restore the survival of the clone [125], while overexpression of oncogenic Ras (*Ras^v12^*) prevents clonal cell death and reverts clones to invasive metastatic tumors [124,125,126,127,128], reminiscent of mammalian cancers. Although the involvement of the endosomal–lysosomal trafficking machinery in the intra-cellular communication and competition within the tumor microenvironment seems probable, such a link has not yet been established experimentally.

Several studies link Dlg, Scrib and Lgl to the EGFR/MAPK pathway, especially in mammalian systems (reviewed in [129,130,131]). Human Scrib (hScrib) binds ERK (via its PDZ1 domain) and anchors it to membrane sites to prevent ERK phosphorylation and inhibit Ras signaling [132]. Scrib can also interact with the EGFR signaling by binding to the ADP-ribosylation factor (Arf)-GAP, GIT1 (G-Protein-Coupled Receptor Kinase-Interacting Protein 1) and βPix (Pak-interactive exchange factor) that acts as a Mitogen-activated protein kinase kinase (MEK)-ERK scaffold [133]. Moreover, mammalian Dlg1 binds MEK2, which phosphorylates and activates ERK [134,135] while Dlg2, Dlg3 and Scrib interact with PP1 phosphatases to downregulate ERK phosphorylation [136].

### 5.3. Interplay of the Dlg-Module with Trafficking and Signaling

Dlg, Scrib and Lgl are involved in vesicle and membrane trafficking in *Drosophila,* yeast and mammals. Dlg and Strabismus (VanGogh) define sites of membrane deposition that allow membrane growth in cellularizing *Drosophila* embryos [137]. In epithelial cells, Dlg and Lgl genetically interact with the Exo84 exocyst complex subunit, required for membrane trafficking and addition [138]. Dlg controls extensive membrane proliferation of the subsynaptic reticulum (SSR) in neuromuscular junctions (NMJs) by binding the t-Soluble NSF attachment Receptor (t-SNARE) protein Syntaxin 18 (also called G-taxin) [139,140]. The yeast Lgl homologues interact directly with the trafficking components Exo84p and Sec9p, while mammalian Lgl binds the t-SNARE protein Syntaxin-4 to direct protein trafficking [112]. Mammalian Scrib regulates exocytosis by binding to the β–Pix-GIT1 complex [133].

Numerous studies support the cooperation of the Dlg-module with vesicle and membrane trafficking, including endocytosis, exocytosis, recycling of endosomes to the cell membrane and retrograde trafficking (and the shuttling between endosomes, biosynthetic or secretory compartments) [98,130,131]. In *Drosophila* sensory organ precursor cells, Lgl controls endocytosis of Sanpodo, a four-pass transmembrane protein and regulator of Notch signaling [141]. In eye epithelia, Lgl attenuates Notch signaling by limiting vesicle acidification [142] and promoting the interaction between the VAMP-associated protein 33kDa (Vap33) and the vATPase complex to inhibit activation of Notch [143]. Lgl localizes on endosomes and *lgl* mutants result in the accumulation of EE (Rab5 and Avl), RE (Rab11) and MVB markers (Hrs) but not LE markers (Car and Rab7), suggesting a role for Lgl in these later stages of endosomal maturation. In this case, Lgl regulates Notch signaling in a Dynamin/Rab5-mediated endocytosis and vesicle acidification but is independent of ESCRT-0 (Hrs) or the Rab11 REs [142]. On the other hand, Scrib was shown to optimize BMP signaling by regulating the basolateral localization of the BMP receptor Thickveins and its internalization in Rab5-positive endosomes in *Drosophila* wing epithelia [144]. Scrib is also required to block the internalization of Eiger, the *Drosophila* homologue of the mammalian Tumor Necrosis Factor (TNF) [145]. Along the same line, mammalian Scrib binds the TSHR (Thyroid-stimulating hormone receptor) to inhibit basal receptor endocytosis and promote its recycling [146].

Very interesting studies have also revealed a role for the Dlg-module in regulating the retromer complex that traffics protein cargo from endocytic vesicles to the trans-Golgi network (TGN) for recycling [147,148]. In MDCK epithelial cells, Scrib negatively regulates retromer-mediated E-cadherin trafficking to the Golgi [148]. In imaginal discs, endocytic itineraries of Crumbs and other retromer-dependent cargo relay on a polarity independent-role of Dlg, Scrib Lgl and AP-2/Dynamin-dependent endocytosis [147]. This post-internalization route, which is independent of the endo-lysosomal transport, recycles retromer-dependent cargoes back to the membrane. This is further supported by the observation that two other components of the retromer sorting complex, Rab9 and Vps29 are severely affected in knockdowns of Dlg-module components while disrupting the retromer components Vps35 and Vps26, enhances the observed Dlg-module phenotypes [147].

## 6. Polarity, Endocytosis and Signaling in Squamous Epithelia: Cell-to-Cell Communication and Coordination in the *Drosophila* Testis

**Figure 3:**
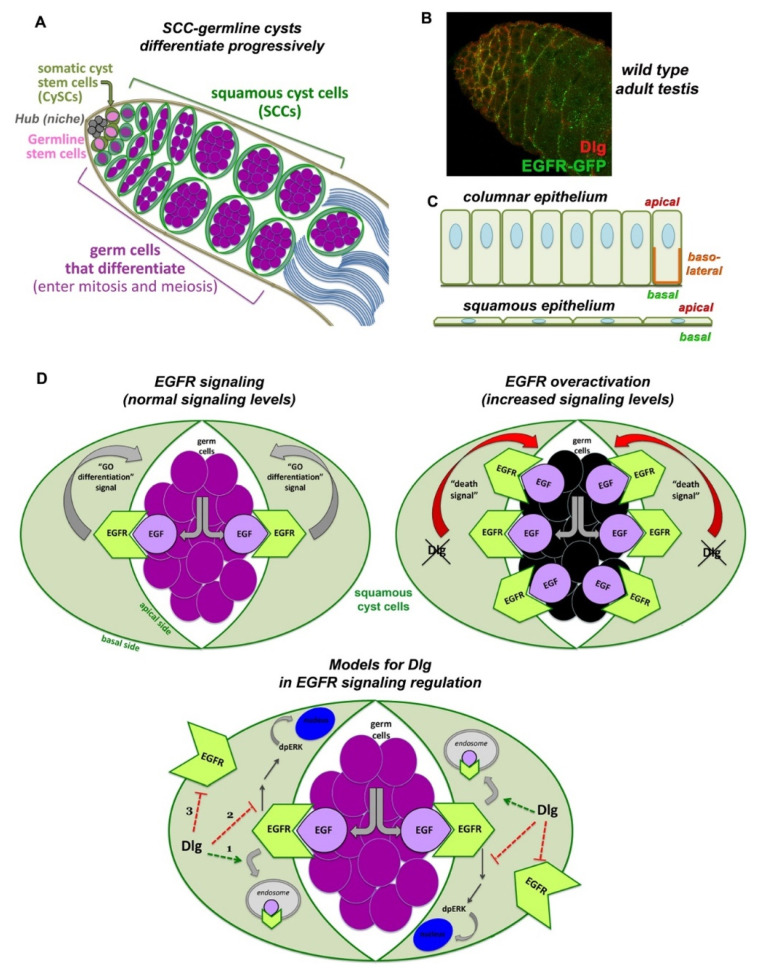
Interplay of Dlg-module, endocytosis and EGFR signaling in squamous cyst cells of the *Drosophila* testis. (**A**) Early spermatogenesis in *Drosophila*. Germline stem cells (GSCs) and somatic cyst stem cells (CySCs) are anchored to the anterior hub cells of the niche (grey). Upon asymmetric division, a GSC gives rise to a distally located daughter germ cell that initiates 4 transit amplifying (TA) mitotic divisions, creating cysts of interconnected spermatogonia. At 16-cell stage spermatogonia exit the mitotic program, turn on the pre-meiotic transcription program, grow enormously and become spermatocytes. CySCs divide giving rise to daughter squamous cyst cells (SCCs), which encapsulate as a pair the germ cells up to sperm individualization. Early SCCs encapsulate spermatogonia, late SCCs encapsulate spermatocytes. (**B**) Dlg (red) and EGFR (green) localize in SCCs. Image frame: 123 μm. (**C**) Columnar epithelia have distinct apical, basal and lateral domains. Squamous epithelial cells lack lateral membranes and show the close proximity of apical vs. basal membranes. (**D**) EGFR in SCCs (green) is activated upon binding of the EGF ligand Spitz, emanating from the germline (purple). Activated EGFR signals back to the germline by sending a (unidentified) “GO differentiation” signal that promotes progressive germline differentiation. Loss of Dlg, Scrib or Lgl function in SCCs, leads to overactivation of the EGFR signaling that sends now a “Death signal” to the germ cells (dying germ cells shown in black). The Dlg-module could attenuate EGFR signaling by (1) cooperating with components of the clathrin-mediated endocytic (CME) pathway, (2) binding and inactivating components of the MAPK/Ras pathway (acting in parallel to the function of CME components), (3) facilitating the polarized distribution of the EGFR on the basal side of SCCs, away from the apical surfaces that face the germ cells secreting the EGF ligand. To simplify the diagram, only Dlg is depicted in (**B**,**C**).

Cells communicate with each other and the local microenvironment by guiding their signaling machineries to discrete cortical membrane domains. Based on the signals they receive, cells adapt their intrinsic features and structural characteristics to achieve a coordinated output and highly ordered organ systems. Thus, elucidating regulatory mechanisms related to cell polarity, endocytosis and signaling, requires a good understanding of apico-basal membrane identities, and the ability to monitor the compartmentation of signaling components, receptors and key regulators with spatial precision. Although this process is straightforward in columnar epithelia, it has not yet been addressed in a systematic way in squamous epithelia (Figure 3C). Squamous epithelial cells are present in several tissues and line the internal body surfaces of many human organs such as the heart, lungs, blood vessels and peritoneal cavity. Mutations in these cells give rise to squamous cell carcinomas [149], all sharing common genetic, histological and signaling defects, including deregulation of receptor tyrosine kinases (RTK). In *Drosophila*, squamous epithelial cells are present in the amnioserosa [150] the follicular epithelium of the ovaries [151,152] and the *Drosophila* testis [153,154].

In the *Drosophila* testis, squamous epithelial somatic cyst cells (SCCs) become unusually thin and elongated by following the growth of the dividing and differentiating germ cells they encapsulate, building together an organoid-like cyst (Figure 3A). Squamous SCCs are also polarized, with apical membranes facing the germline and basal membranes facing the outside of the cysts (Figure 3C). They actively support the progressive steps of germ cell differentiation through increasing levels of the Epidermal Growth Factor (EGFR) activation [155] that localizes in SCCs (Figure 3B). EGFR is activated by the binding of the EGF ligand Spitz, secreted from the germ cells (sSpi). The protease Stet cleaves Spitz, which can bind the EGFR on SCC and activate the Ras/MAPK signaling pathway, leading to the double phosphorylated MAPK *rolled* (dpERK) entering the nucleus [156]. This first step of differentiation is required for the germ cells to properly enter and execute the transit-amplifying (TA) program of synchronous mitotic divisions [155,156,157,158,159]. In the absence of EGFR-derived signals, germ cells cannot differentiate and overproliferate as stem cell-like germ cells [157,158], while increased EGFR signals induce germ cell death (Figure 3D) [153]. Higher levels of EGFR activation in cyst cells are required for spermatogonia to end the TA divisions and initiate the spermatocyte pre-meiotic program [155]. Presumably, EGFR effects are achieved in a dose-dependent manner, explaining why fine-tuning of signaling levels is crucial for stepwise germline differentiation. However, the way squamous SCCs coordinate endocytosis and polarity with EGFR signaling levels, and potentially adapt them to their thin elongated morphology has been largely unknown [154].

A recent study revealed that the cortical polarity proteins Dlg, Scrib, Lgl and clathrin-mediated endocytosis (CME) downregulate EGFR signaling levels in SCCs [153] to prevent germ cell death and promote the differentiation of the germline into mature sperm (Figure 3D). These proteins are known to establish and maintain architecture and homeostasis in the *Drosophila* male gonads and larval testes [160,161,162,163,164]. More precisely, somatic cells fail to extend projections and encapsulate the germ cells in embryonic gonads of male *scrib* or *dlg* mutant flies, suggesting a role in establishing intimate soma-germline contacts [160,164]. Knockdown of *dlg*, *scrib*, *lgl* or CME components (Clathrin heavy chain, AP-2/Adaptin and Dynamin/Shibire) specifically in the squamous SCCs, mimics the effect of EGFR overactivation, resulting in germ cell death and increased signal transduction via the EGFR. Lowering EGFR or downstream signaling components rescues those defects [153]. Interestingly, membrane PIP2 has emerged as a critical regulator that contributes to EGFR/Ras-mediated activation of the MAPK in the SCCs in the system, suggesting a new link between polarity, endocytosis and EGFR/MAPK signaling [153].

Yet, the exact mechanism of how polarity and endocytosis regulate EGFR signaling levels under physiological conditions in the *Drosophila* testis and how EGFR is distributed to apical vs. basal cyst cell membranes is not yet established. Dlg, Scrib or Lgl could act to attenuate EGFR signaling in SCCs by potentiating endocytosis of the EGFR receptor together with CME. Alternatively, the Dlg module could attenuate EGFR/MAPK signaling independent of endocytosis, by binding and inactivating signal transduction components downstream of the EGFR (Figure 3D), as in other tissues (outlined in Section 5.2) [132,133,134,135]. An alternative possibility could be that Dlg, Scrib and Lgl regulate the polarized distribution of the EGFR by directing the receptor to the basal SCC membranes and attenuate the EGFR signaling by preventing its access to Spitz from the germline. This latter mechanism, of silencing the EGFR through basal or basolateral trafficking, has been shown in other polarized epithelia as well (outlined in Section 3) [57,58,59].

**Figure 4:**
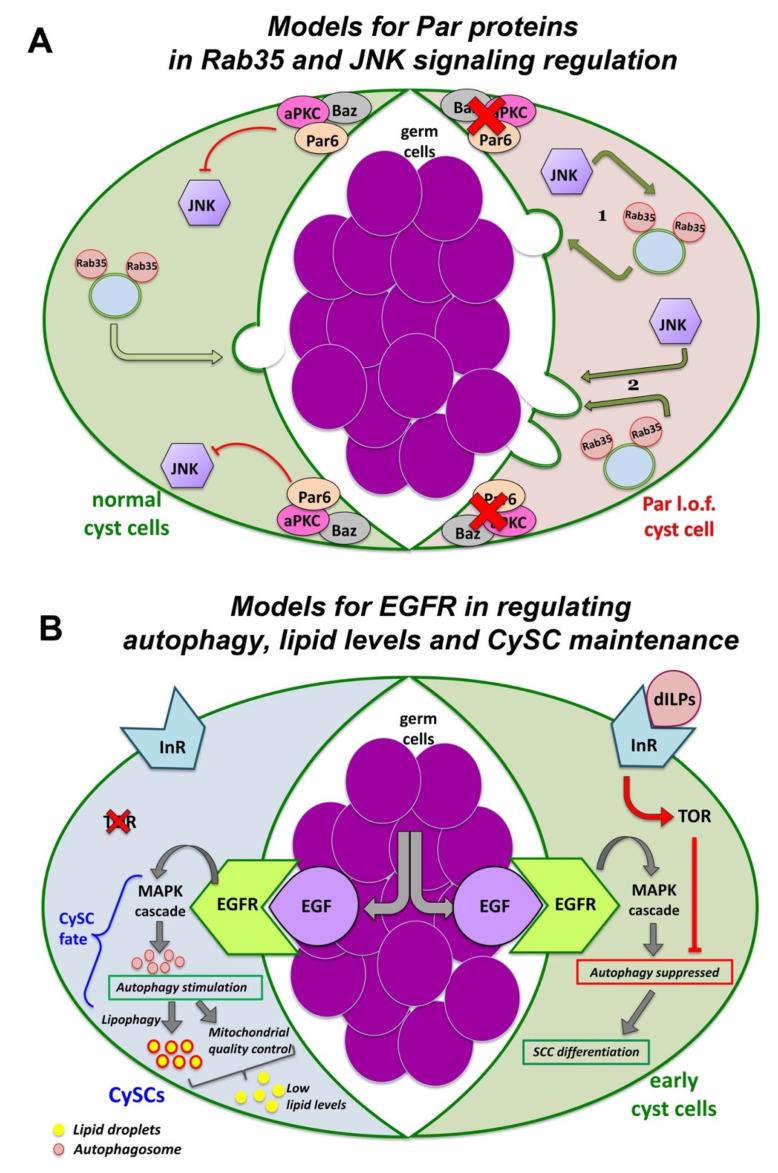
Pathways of signaling, trafficking and autophagy in squamous cyst cells (SCCs) of the *Drosophila* testis. (**A**) PAR components suppress JNK signaling in SCCs. In normal (wild type) SCCs, the PAR complex reduces JNK pathway activation (left SCC shown in green). In PAR loss of function (l.o.f.) SCCs (right SCC shown in red), de-repression of JNK signaling leads to spermatocyte death. JNK may act (1) by cooperating with Rab35 to deliver the death signal to the germline or (2) independently (but in parallel to Rab35) to induce spermatocyte phagocytosis by the SCCs. (diagram adapted with modifications from Ref. [166]). (**B**) EGFR stimulates autophagy to control lipid levels and CySC maintenance. In CySCs, binding of the EGF ligand Spitz to the EGFR activates the MAPK signaling cascade and stimulates autophagy. Autophagy prevents the accumulation of lipids, which would otherwise compromise GSC homeostasis and survival. Lipophagy and mitochondrial quality control (through fatty acid oxidation) further reduce lipid accumulation. In early-stage SCCs, TOR via the ILR-PI3K-TOR pathway, suppresses autophagy temporarily and allows SCC differentiation to proceed (diagram adapted with modifications from Refs. [167,168]). To simplify the diagrams, two different cyst cells are depicted in (**A**,**B**) as part of the same cyst cell-germline cyst.

Interestingly, the function of Dlg, Scrib and Lgl in SCCs is independent of the apical cyst cell polarity and SJ-mediated barrier function that seals each SCC-germline cyst. Knockdown of apical PAR-complex determinants, e.g., Bazooka (Baz/Par3) [165,166] or of septate junction (SJ) core components in SCCs result in distinct phenotypes to the Dlg-module, while the barrier function is largely unaffected [153]. Interestingly, Baz/aPKC/Par6 apical polarity complex in SCCs is required for the survival of the pre-meiotic germ cells they enclose, called spermatocytes (Figure 4A; right side). Loss of function of any of these components in SCCs results in overactivation of JNK signaling that mediates the germ cell death via the recycling endosome small GTPase Rab35 [166]. The exact nature of this mechanism, i.e., whether JNK and Rab35 act cooperatively or independently but in parallel ways, to promote germ cells death is so far unclear (Figure 4A; left side).

Besides its role in regulating SCC-germline communication and co-differentiation in the *Drosophila* testis, the EGFR stimulates basal autophagy to maintain the identity of the somatic cyst stem cells (CySCs) and to control the lipid levels through lipophagy (the autophagy induced breakdown of lipid droplets) (Figure 4B; right side) [167,168]. CySCs encapsulate the germline stem cells (GSCs) attached to the niche cells of the hub (Figure 3A). When CySCs divide asymmetrically, their daughter SCCs move away from the hub to initiate their own differentiation. Moving away from the hub, early-stage SCCs experience a burst of insulin signaling, via the Insulin-Like Receptor-PI3K-mTOR (mammalian target of rapamycin/mTOR) pathway, which suppresses autophagy temporarily and allows SCC differentiation to proceed (Figure 4B; left side). Defective autophagy results in lipid accumulation, increased CySCs numbers and loss of early SCCs [167,168]. Therefore, fine-tuning of EGFR-mediated autophagy is necessary for the proper control of CySC maintenance vs. SCC differentiation fate in the *Drosophila* testis, which in turn safeguards germline differentiation, as discussed previously [167,168]. Uncovering the mechanisms that govern EGFR-mediated autophagy may prove valuable to understanding autophagy misregulation in cancers and how adapted autophagy affects resistance to anti-EGFR monoclonal antibody treatments [169,170,171].

A screen in *Drosophila* testis SCCs provided more insights on the importance of endocytic components in SCC signaling regulation. For example, the early endosome GTPase Rab5 is critically required for proper SCCs and germline differentiation. Knockdown of Rab5 in SCCs results in tumor-like overgrowths with (1) germ cells that become arrested at the spermatogonial-stage (the mitotic division stage) and (2) SCCs that show elevated levels of ectopic Hedgehog (Hh) and Janus kinase signal transducer and activator of transcription (JAK-STAT) activation, increased BMP ligands and an expansion of the Zfh-1 marker (normally present in CySCs and early SCC) in SCCs away from the niche [165]. Thus, Rab5 normally acts to downregulate stem cell maintaining signals in SCCs as they move away from the niche, and thereby allows spermatogenesis to progress beyond the spermatogonial stage [165].

Taken together, the *Drosophila* testis with the squamous epithelial cyst cells and the cross-communication to the encapsulating germ cells, have provided new insights into the dynamic regulation of a wide range of endosomal–lysosomal trafficking components with short-range signaling and cortical polarity cues during spermatogenesis.

## 7. Emerging Themes in other Tissues and Cellular Contexts

### 7.1. The Drosophila Gut and Epithelial Homeostasis

**Figure 5:**
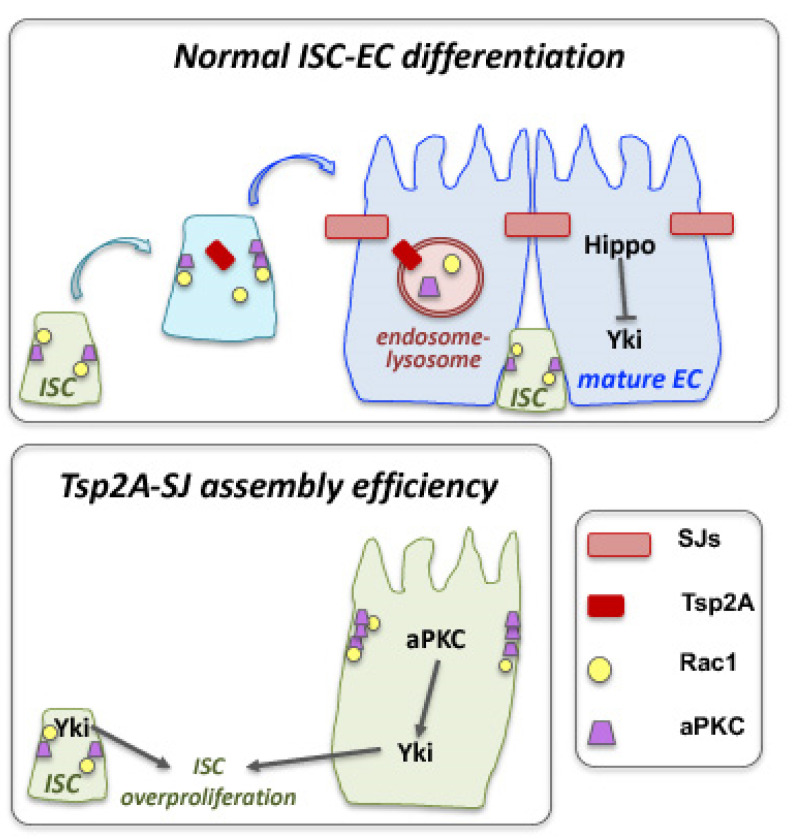
Crosstalk of septate junctions and cell compartmentation with signaling and fate determination in the *Drosophila* intestine. The septate junction (SJ) and transmembrane protein tetraspanin 2A (Tsp2A) plays a critical role in the differentiation of intestinal stem cells (ISCs) to adapt the absorptive enterocyte (EC) fate, by promoting SJ assembly and modulating the function of the atypical protein kinase C (aPKC). The presence of a functional Tsp2A in differentiating ISCs promotes the degradation of aPKC, which antagonizes the Hippo pathway. This permits Hippo activation that inactivates Yki. Loss of Tsp2A, prevents SJ formation, affects the localization of adherens junction components and aPKC leads to Yki hyperactivation and overproliferation of ISC cells (diagram adapted with modifications from Ref. [174]).

In the *Drosophila* intestine, asymmetric division of intestinal stem cells (ISCs) gives rise to a new ISC and a daughter progenitor that becomes either an enteroblast (EB) that will differentiate into enterocytes (EC, 90%) or an enteroendocrine precursor (EEP) that will differentiate into secretory enteroendocrine cells (EE, 10%) [172,173]. This fate decision relays on the Notch signaling pathway. Although EGFR/Ras/MAPK, JAK-STAT and the Hippo pathway are known to regulate ISCs, little is known about the inputs that modulate their activities. In this epithelium, adherens junctions (AJs) are seen in ISCs and EBs, while septate junctions (SJs) in ECs. SJ assembly occurs during ISC-EC differentiation, and the transmembrane scaffolding protein tetraspanin 2A (Tsp2A) is critically required for SJ assembly [174]. Under normal conditions, Tsp2A undergoes internalization and thereby mediates the degradation of atypical protein kinase C (aPKC), which antagonizes the Hippo pathway (Figure 5). This permits Hippo activation, which can now restrict ISC proliferation by turning off Yorkie (Yki)-JAK-STAT activity. Tsp2A knockdown causes (1) defects in the localization of other SJ proteins and SJ assembly, (2) increased expression of the AJ protein Armadillo (Arm) and the basolateral protein Lgl, as well as (3) accumulation of aPKC and Yki hyperactivity. Thus, SJ assembly is pivotal for EC differentiation, while Tsp2A endocytosis has a key physiological role in restoring the Hippo pathway and restricting proliferation after tissue repair [174].

Another regulatory mechanism was revealed by analyzing sorting Nexins (SNXs), an evolutionarily conserved family of proteins that regulate intracellular membrane traffic most likely through their phosphoinositide-binding domain [175]. In the *Drosophila* midgut, the nexin SH3PX1 was shown to be critical for restraining ISC proliferation by downregulating the EGFR-Ras-MAPK signaling (highly expressed in ISCs), through an endocytosis-autophagy network [175]. This network includes Dynamin, Rab5, Rab7, Autophagy proteins (Atg1, 5, 6, 7, 8a, 9, 12, 16) and the Syntaxin Syx17. Blocking autophagy (or components in that network) stabilizes ligand-activated EGFR and directs them via the Rab11-recycling endosomes back to the plasma membrane, leading to increased ERK and Ca^2+^ signaling, ISC mitotic divisions and overproliferation. Thus, intracellular vesicle trafficking through the endocytosis-autophagy pathway can restrain ISC proliferation and hyperplasia by counteracting the EGFR activity. This mechanism is critical to maintain gut epithelial homeostasis and can provide an alternate route to EGFR signaling activation in human cancers [175].

RAL small GTPases are effectors of Ras signaling, widely expressed in the intestinal epithelium [176,177]. They activate the Wingless-integrated (Wnt) pathway in ISCs through the internalization of Wnt receptors from the plasma membrane. This is important for ISC numbers and intestine regeneration following damage [176]. In parallel to Wnt, RAL GTPases also activate the EGFR/MAPK signaling in the intestine by promoting EGFR internalization. Knocking down *Drosophila* RalA from the intestinal stem and progenitor cells in *Drosophila* leads to increased levels of plasma membrane-associated EGFR and decreased MAPK pathway activation. Thus, in contrast to other systems where receptor endocytosis attenuates signaling [15,28,29], here, Wnt and EGFR signaling is not limited to the plasma membrane since internalization seems to amplify the signaling activity [176,177].

### 7.2. Tissue Morphogenesis and Remodeling

Endocytosis plays an important role in morphogenetic changes of lateral membrane shortening. In the *Drosophila* ovary, follicle cells undergo a cuboidal-to-squamous transformation, which involves lateral membrane shortening and apical membrane extension via the action of the Tao protein [151]. Tao normally reduces lateral adhesion between epithelial cells by promoting endocytosis of the transmembrane adhesion protein Fasciclin II (Fas II), involved in homophilic interactions. Mutations in the *tao* gene, result in Fas II accumulation on the lateral membrane of those cells that block cuboidal-to-squamous cell remodeling [151]. This study links the importance of how endocytosis is downregulating adhesion molecules to achieve the plasticity that will allow morphogenetic changes to proceed [152].

During *Drosophila* gastrulation and epithelial morphogenesis, endocytosis and plasma membrane compartmentation differentially regulate Rho1 and Myosin II activation [178]. The GPCR protein Smog normally concentrates on the apical surface of ectodermal cells, as well as the membrane invaginations that these cells have. Upon binding to its ligand Folded gastrulation (Fog), Smog forms homo-clusters, and endocytosis follows through the action of G protein-coupled receptor kinase 2 (Gprk2) and β-Arrestin-2 kinases. Low Fog concentration induces very low Rho1 signaling, as Gprk2 and β-Arrestin-2 attenuate Rho1 signaling by regulating Smog endocytosis. However, when Fog concentration is high, endocytosis is reduced, and Smog accumulates in localized apical plasma membrane invaginations that act as signaling centers and induce high levels of Rho1/Myosin II activation [178]. In the mesoderm, Smog accumulates in larger, more numerous, apical plasma membrane invaginations, and it displays larger Smog homo-clusters compared to the ectoderm. This dynamic partitioning of active Smog in oligomer clusters has a direct impact on signaling levels that transform the apical membrane invaginations into compartmentalized signaling centers [178], which can drive cell contractility and constrictions that characterize gastrulation morphogenetic events.

### 7.3. Drosophila Nephrocytes: An Emerging Model in Apical-Basal Polarity and Endocytosis

Apical-basal polarity components play a key role in endocytosis and cellular trafficking in *Drosophila* nephrocytes, the equivalent of mammalian podocytes, as part of the filtration barrier in human kidneys [179,180,181]. Nephrocytes develop from myoblasts and are found in two populations floating in the hemolymph of larval and adult flies: (1) the garland nephrocytes, bi-nucleated cells that surround the connection site of the esophagus and the proventriculus; and (2) the pericardial nephrocytes, mono-nucleated cells that line up along the heart tube [179,180,182]. Both cell types possess similar ultrastructural and functional characteristics. Yet, unlike mammalian podocytes, *Drosophila* nephrocytes are not physically connected to the fly excretory and osmoregulatory system of the Malpighian tubules (the equivalent of mammalian liver and kidneys). Fly nephrocytes filtrate the hemolymph and endocytose proteins and toxins to store and permanently inactivate them [179,180,182].

**Figure 6:**
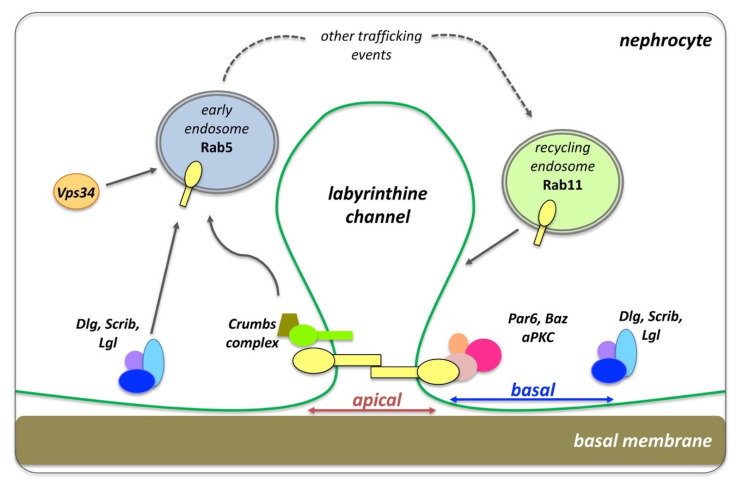
The slit diaphragm (SD), endocytosis and polarity establishment in *Drosophila* nephrocytes. The apical polarity Crumbs and PAR complexes localize at the SD, which corresponds to the apical region of the nephrocytes. Dlg, Scrib and Lgl localizes to the basal side the nephrocytes, flanking neighboring SDs. Cell trafficking, including early endosomes and recycling endosomes, are critical for the function of the SD and proper endocytic function (diagram adapted with modifications from Ref. [179]).

Although nephrocytes are not of epithelial origin, they resemble epithelial cells as they are polarized. Nephrocytes have an enlarged apical domain and reduced basal surface (lacking lateral sides), which altogether build a cell surface comprised of membrane invaginations that form finger-like labyrinthine channels [179] (Figure 6). The slit diaphragm (SD) establishes the filtration barrier that spans the opening of these labyrinthine, where hemolymph proteins get reabsorbed via endocytosis. Many components of the SD have orthologs in mammals, such as Sticks and stones (Sns), the *Drosophila* homologue of the mammalian Nephrin. Apical polarity components of the PAR and Crumbs complexes localize and build the SD, while basolateral components of the Dlg-module localize at the basal region between neighboring SDs. Clathrin-mediated endocytosis (CME), Rab5-dependent EE, Rab11-dependent RE and the exocyst complex are critical for SD maintenance [179,180,181] (Figure 6).

A recent study [180] has shown that knockdown of PAR complex proteins results in severe endocytosis and SD defects, associated with problems in atypical protein kinase C (aPKC) membrane targeting. Loss of basolateral polarity regulators *dlg, scrib* and *lgl* disrupt the localization of Sns on SDs, yet severe defects on nephrocyte endocytic function are seen only in *scrib* loss of function nephrocytes [180]. Crumbs, via its FERM-domain, also regulates nephrocyte SD assembly and endocytosis, while Moesin binds the Crumbs FERM domain to support the endocytic function [182]. Another study [181] confirmed that basolateral polarity proteins work together with apical components to promote the endocytic trafficking of SD proteins in nephrocytes. More precisely, loss of *dlg, scrib, lgl* and *par1* function in nephrocytes, led to the accumulation of SD proteins in Rab5 vesicles which mis-localized, while the integrity of SDs was compromised. Moreover, Vps34 (PI3K59F) has been shown to regulate autophagosome-autolysosome formation in nephrocytes, associated with the accumulation of Rab5 and Rab7 endocytic compartments, suggesting a disruption in vesicular transport [183]. Furthermore, loss of the membrane PI(4,5)P2 from the SD region leads to a significant reduction in endocytosis and loss of SD integrity. Conversely, increased levels of the phosphatidylinositol(3,4,5)-trisphosphate (PI(3,4,5)P3) give rise to a stronger SD via ectopic activation of the Akt/mTOR pathway, but this is still linked to the endocytosis defects [184]. These studies reveal how apical-polarity determinants set the stage for proper establishment, integrity and function of SDs in nephrocytes, which in turn maintain endocytosis and promote nephrocyte homeostasis.

### 7.4. The Role of Adaptor Proteins and Endocytosis in Nutrient-Based Decision-Making: Examples from the Budding Yeast

In multicellular organisms discussed so far, the interplay of cellular trafficking, polarity and signaling has been actively involved in developmental and morphogenetic decisions that shape tissues and organs. In unicellular organisms such as the budding yeast *Saccharomyces cerevisiae*, adaptor proteins and signaling components work together to regulate the endocytosis of nutrient transporters and thereby modify the yeast’s growth and response to environmental cues [185,186,187]. Nutrient-importing transporter proteins or permeases (e.g., for amino acids and glucose) embedded in the plasma membrane of *S. cerevisiae* are destined to switch in order to match the food availability. This is performed through the membrane addition of the necessary transporters through the secretory pathway and the endocytic removal of those no longer required. For example, yeast growing in a lactate medium would favor the addition of the Jen1 transporter that absorbs this molecule, while the addition of glucose would promote endocytosis of Jen1 and its replacement with a glucose transporter [185,186,187]. Unneeded transporters are triggered for endocytic removal by ubiquitination through the Nedd4 ubiquitin ligase Rsp5, while Arrestin-Related Trafficking adaptor (ART) proteins (of the alpha-Arrestin family) act as adaptors for Rsp5. *S. cerevisiae* has 14 different ARTs, which are under multilevel control of the major nutrient-sensing systems and become targeted by signaling pathways [185].

The target of rapamycin complex 1 (TORC1) signaling is one of the critical signaling pathways that regulate ART proteins [185]. TORC1 stimulates cell growth by promoting anabolic processes and repressing catabolic processes. TORC1 has been shown to modulate nutrient uptake by affecting the composition of plasma membrane amino acid transporters, by regulating ubiquitin-mediated endocytosis [188]. TORC1 negatively regulates the Npr1 kinase, which can inhibit Art1activity by preventing its phosphorylation. In this way, Npr1 prevents the Art1-mediated targeting of Rsp5 and maintains the abundance of amino acids transporters at the plasma membrane [188].

Distinct ART–Rsp5 complexes can act at different yeast internal compartments to regulate the fate of nutrient transporters along the endocytic pathway [185]. Such an example is the ART proteins Art-1 and Bul1/2, which regulate the endocytosis of the arginine permease Can1 in different ways. Activation of Art1 by TORC1 in the presence of the arginine substrate triggers ubiquitination and a conformational change in Can1 (from the outward open to the inward open state) [189] that recycles Can1 back to the plasma membrane, via the trans-Golgi network (TGN). When TORC1 is hyperactivated in the absence of arginine in the medium, it is the Bul1/2 that promotes Can1 endocytosis and targets it to degradation through vacuolar sorting [190]. This is an example of how nutrient-sensing can activate distinct ARTs to control the ubiquitin- and endocytic-dependent downregulation of nutrient transporters.

Excess of individual nutrients or their deprivation can activate complementary ART-Rsp5-complexes to control selective endocytosis and adapt nutrient acquisition. Art1–Rsp5 not only promotes endocytosis of the Can1 arginine transporter, but it also mediates the endocytosis of the methionine transporter Mup1 and the lysine transporter Lyp1 in response to the excess of their respective amino acid substrates [191]. Conversely, upon amino acid and nitrogen starvation, the same set of amino acid transporters are down-regulated by Art2–Rsp5 [185,191]. Glucose deprivation regulates Art8 activation via ubiquitination to trigger glucose transporter endocytosis, while glucose-induced deubiquitylation of Art8 correlates with its phospho-dependent association to 14-3-3 proteins [186]. These data illustrate novel mechanisms by which nutrients modulate ART adaptor activity and endocytosis.

Post-translational modification of ART proteins regulates their switch from active to inactive state. Rod1/Art4 is an ART protein necessary for yeast cells to endocytose the lactate transporter Jen1 in the presence of glucose. Yeast cells preferentially use glucose as an energy nutrient over lactate when possible. Rod1 phosphorylation is regulated by glucose availability, which consequently modulates its activity. More precisely, glucose-induced dephosphorylation of Rod1 promotes its ubiquitylation, a modification that is essential for Jen1 endocytosis [187]. The fine balance of Rod1 post-translational modifications (dephosphorylation vs ubiquitylation) is coordinated by a phospho-dependent interaction of Rod1 with yeast 14-3-3 proteins, which inhibits Rod1 ubiquitylation. Upon loss of Rod1, cells cannot ubiquitinate Jen1, suggesting that Rod1 helps Rsp5 ubiquitinate Jen1. A more recent study has further shown that Rod1 modulates the post-endocytic sorting of Jen1 to the yeast lysosome [192]. The role of Rod1 in glucose-induced transporter endocytosis provides an interesting molecular mechanism of an ARF-dependent adaptor activation in response to intracellular signaling [187,192].

## 8. Conclusions

Trafficking through the endo–lysosomal system is intimately linked to polarity and signaling, and together they play a central role in regulating fundamental aspects of cell physiology and homeostasis. Research over recent decades revealed the complexity of their cooperation and mutual dependence across multiple pathways where trafficking, polarity and signaling intersect. Along this line, polarity scaffolds are not just static barriers and endosomal compartments are not simple cargo transporters. Compelling evidence shows that they emerge as dynamic organizing centers involved in site-specific protein targeting (or exclusion) and in stabilizing supramolecular signaling complexes that (1) provide guiding cues for targeted membrane insertion, cell compartmentation, communication, and remodeling; and (2) actively control the timing, amplitude and specificity of intracellular signaling.

*Drosophila*, which combines a relatively simple organization with sufficient cellular complexity and conservation, allowed us to “zoom” into different cell types and uncover the regulatory strategies in very diverse contexts. From gut intestinal epithelia, sensory organ precursors (SOPs) and imaginal epithelia of developing wings, legs and ommatidia, to squamous epithelia in testis and ovaries, and non-epithelia nephrocytes, research in the fruit fly has made a major contribution to our understanding of how endosomal–lysosomal processes, polarity and (intracellular and inter-cellular) signaling, coordinate cellular responses and establish homeostasis. Although we are still far from breaking the mysteries of endocytosis across species [193], we can appreciate the sophisticated, multiple levels of control that are necessary to orchestrate the formation of functional tissues and organs.

## Data Availability

Not applicable.

## Abbreviations

clathrin-mediated endocytosis (CME), clathrin independent endocytosis (CIE), early endosome (EE), recycling endosome (RE), multi-vesicular bodies (MVB), late endosomes (LE), trans-Golgi network (TGN), intra-luminal vesicles (ILV), endosomal sorting complex required for transport (ESCRT), Phosphatidylinositol 4,5-bisphosphate [PtdIns(4,5)P2] (PIP2), atypical protein kinase C (αPKC), Hedgehog (Hh), Janus kinase signal transducer and activator of transcription (JAK-STAT), Jun N-terminal kinase (JNK), Receptor tyrosine kinase (RTK), Epidermal Growth Factor Receptor (EGFR), ubiquitinated EGFR (EGFR-Ub), de-ubiquitinating enzyme (DUB), Spitz (Spi), Discs large (Dlg), Scribble (Scrib), Lethal (2) giant larvae (Lgl), adherens junction (AJ), septate junctions (SJs), somatic cyst cells (SCCs), somatic cyst stem cells (CySCs), germline stem cells (GSCs), mammalian target of rapamycin (mTOR), target of rapamycin complex 1 (TORC1).
